# Correction: Davidi et al. Tumor Treating Fields (TTFields) Concomitant with Sorafenib Inhibit Hepatocellular Carcinoma *In Vitro* and *In Vivo*. *Cancers* 2022, *14*, 2959

**DOI:** 10.3390/cancers15041182

**Published:** 2023-02-13

**Authors:** Shiri Davidi, Sara Jacobovitch, Anna Shteingauz, Antonia Martinez-Conde, Ori Braten, Catherine Tempel-Brami, Einav Zeevi, Roni Frechtel-Gerzi, Hila Ene, Eyal Dor-On, Tali Voloshin, Itai Tzchori, Adi Haber, Moshe Giladi, Adrian Kinzel, Uri Weinberg, Yoram Palti

**Affiliations:** 1 Novocure Ltd., Haifa 3190500, Israel; 2 Novocure GmbH, 81925 Munich, Germany

The authors wish to make minor corrections to Figure 1 and Figure 2 of the following paper [1], as detailed below:

On page 6, the Y axis of Figure 1c was not correct. The corrected Figure 1 appears below.

**Figure 1:**
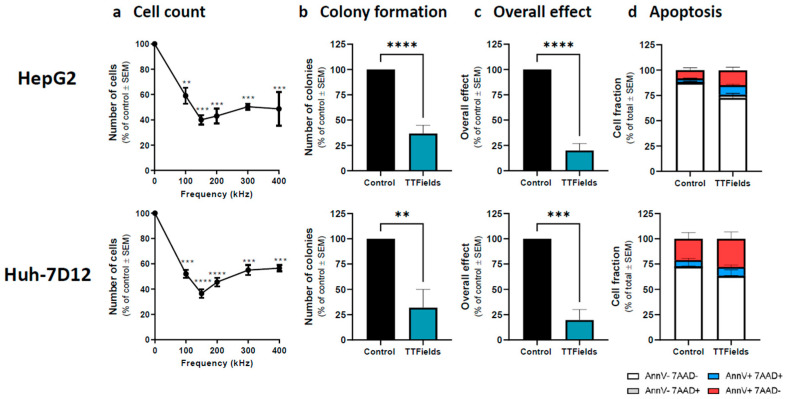
Efficacy of TTFields for treatment of HCC cells. HepG2 and Huh-7D12 cells were treated with TTFields (1.0 and 1.7 V/cm RMS, respectively) for 72 h. Cell counts were determined following treatment with TTFields at a frequency range of 100–400 kHz (**a**). Values are mean (N ≥ 3) ± SEM. ** *p* < 0.01, *** *p* < 0.001, and **** *p* < 0.0001 relative to control; one-way ANOVA. Clonogenicity (**b**), overall effect (**c**), and apoptosis (**d**) were examined for treatment of the cells with 150 kHz TTFields. Values are mean ± SEM. ** *p* < 0.01, *** *p* < 0.001, and **** *p* < 0.0001 relative to control; For apoptosis assay live cells: *p* < 0.05 for HepG2 and *p* = 0.14 for Huh-7D12 relative to control; Student’s *t*-test. ANOVA = analysis of variance; HCC = hepatocellular carcinoma; RMS = root mean square; SEM = standard error of the mean; TTFields = Tumor Treating Fields.

On page 7, the color coding was missing in Figure 2. The corrected Figure 2 appears below.

**Figure 2:**
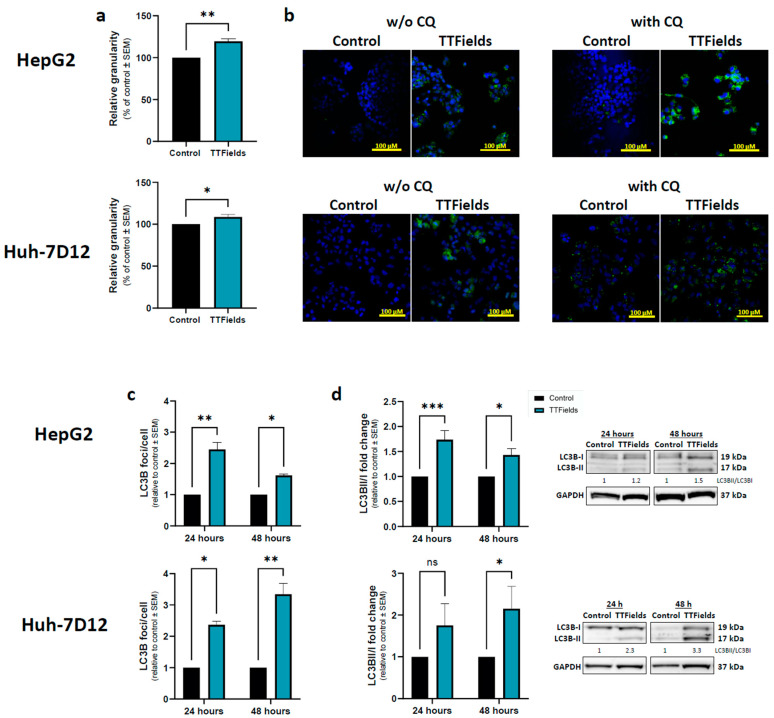
Effects of TTFields on autophagy levels in HCC cells. HepG2 and Huh-7D12 cells were treated with 150 kHz TTFields (1.0 and 1.7 V/cm RMS, respectively) for 72 h, and median side-scatter values were measured as representing changes in cellular granularity (**a**). Cells treated for 48 h were examined for LC3 foci formation by fluorescent microscopy, with or without addition of CQ during the last 3 h of the experiment (**b**), LC3 in green staining and DAPI in blue. Experiments performed with 24 or 48 h application of TTFields with addition of CQ during the 3 final hours of the experiments were quantified for LC3 foci formation by immunofluorescence (**c**), and for LC3-II to LC3-I ratio by immunoblotting (**d**). Values are mean (N ≥ 3) ± SEM. * *p* < 0.05, ** *p* < 0.01, and *** *p* < 0.001, relative to control; two-way ANOVA. ANOVA = analysis of variance; CQ = chloroquine diphosphate; DAPI = 4′,6-diamidino-2-phenylindole; HCC = hepatocellular carcinoma; ns = non-significant; RMS = root mean square; SEM = standard error of the mean; TTFields = Tumor Treating Fields.

The corrections do not affect the main scientific results and the final conclusions of this manuscript. The authors would like to apologize for any inconvenience caused. The original article has been updated.
