# Supplementary material for: Tumor Treating Fields (TTFields) Concomitant with Sorafenib Inhibit Hepatocellular Carcinoma In Vitro and In Vivo
Source: Cancers (Basel). 2022 Jun 15;14(12):2959. doi: 10.3390/cancers14122959 (PMC9220990; doi:10.3390/cancers14122959)

Figure S1. Mechanism of TTFields in combination with sorafenib in Huh-7D12 cells.
Huh-7D12 cells were treated for 6, 24, or 48 hours with 150 kHz TTFields, 3 μM sorafenib, or the two treatments combined, followed by Western blot examination of the autophagy markers beclin-1 and LC3 (a), the ER stress marker GRP78 (b), and the apoptosis marker cleaved PARP (c).
Values are mean ($N \geq 3$) ± SEM. *$p < 0.05$, **$p < 0.01$, ***$p < 0.001$, and ****$p < 0.0001$ relative to time-respective control; two-way ANOVA.
ANOVA = analysis of variance; SEM = standard error of the mean; TTFields = Tumor Treating Fields.

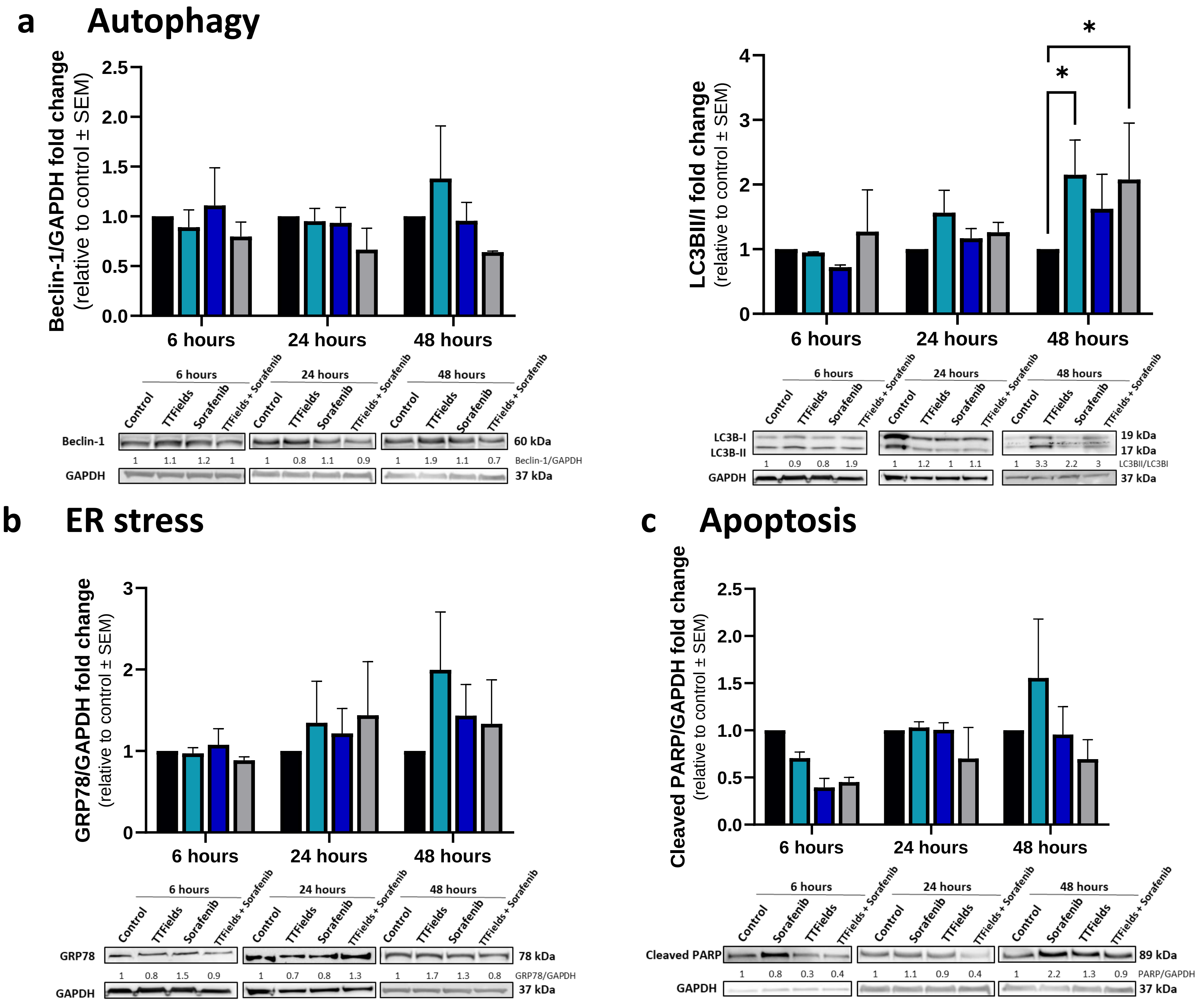

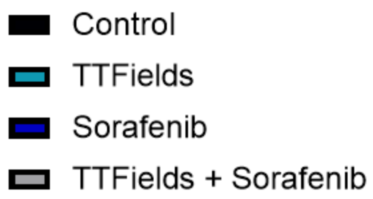

Figure S2. TTFields frequency scan in rat N1S1 HCC cells.
N1S1 cells were treated with TTFields (1.7 V/cm RMS) across a frequency range of 100–400 kHz, and cell count were determined following 24 hours of treatment.
Values are mean (N ≥ 3) ± SEM. *p < 0.05 relative to control; one-way ANOVA.
ANOVA = analysis of variance; HCC = hepatocellular carcinoma; RMS = root mean square; SEM = standard error of the mean; TTFields = Tumor Treating Fields.

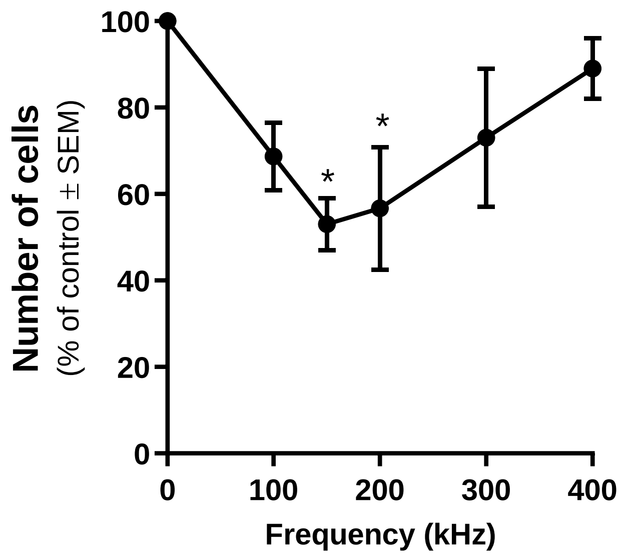

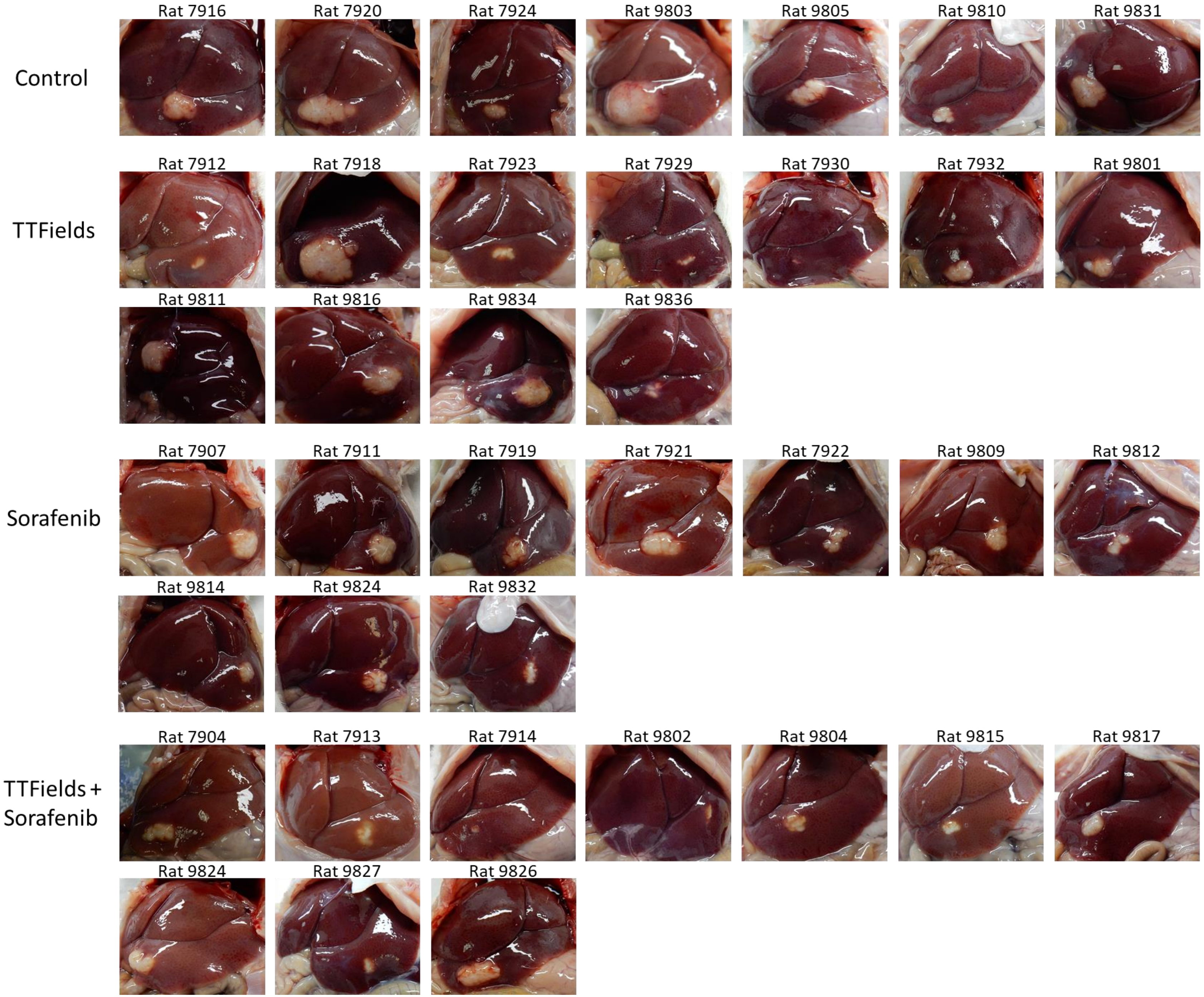

Figure S3. Images of tumors within the livers of the rats treated with sham heat (control rats), TTFields, sorafenib, or TTFields plus sorafenib.

Figure 3.

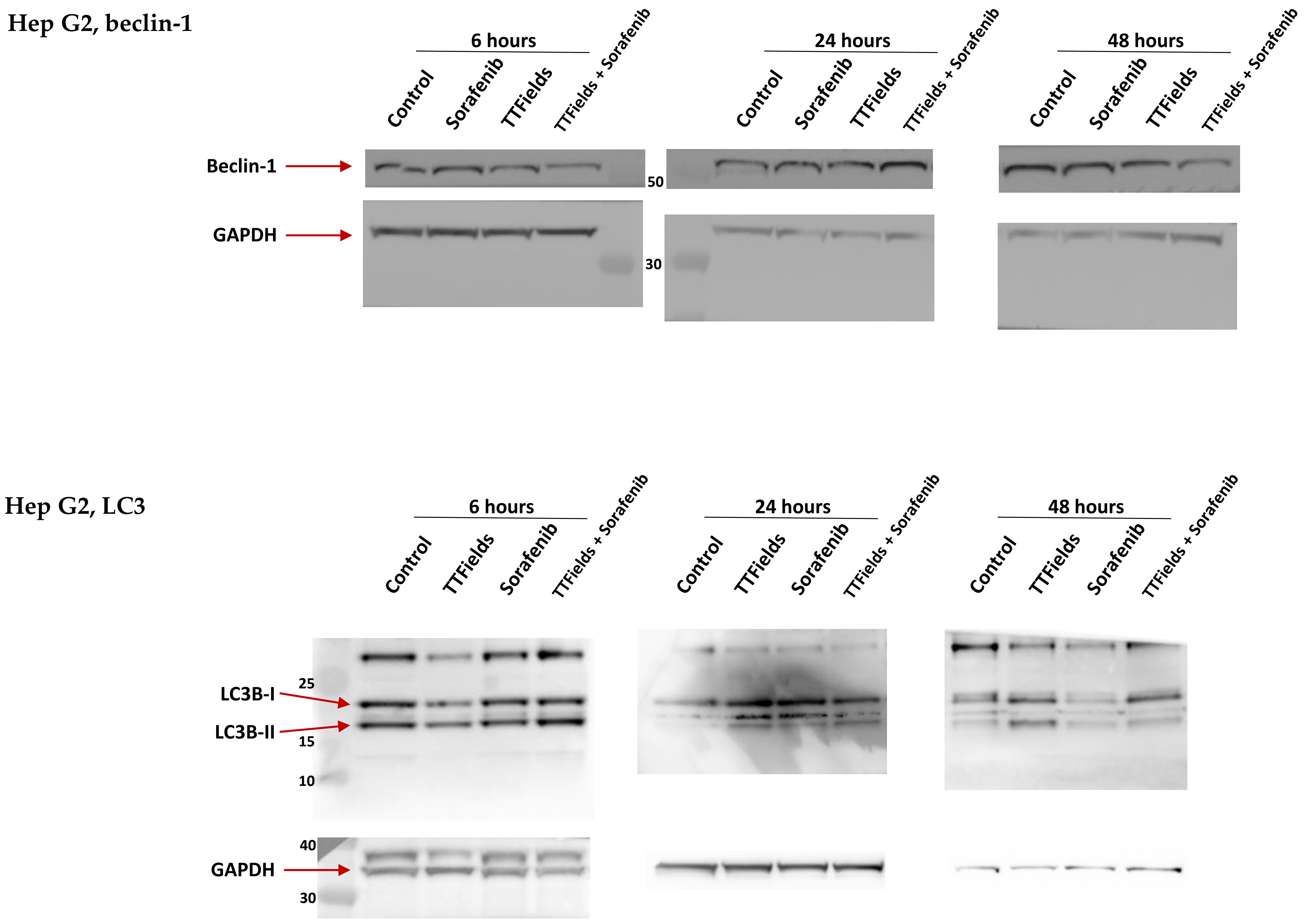
Hep G2, beclin-1
6 hours
24 hours
48 hours
Control
Sorafenib
TTFields
TTFields + Sorafenib
Beclin-1
GAPDH
50
30
Hep G2, LC3
Control
TTFields
Sorafenib
TTFields + Sorafenib
LC3B-I
LC3B-II
25
15
10
40
30
GAPDH

Figure 3.

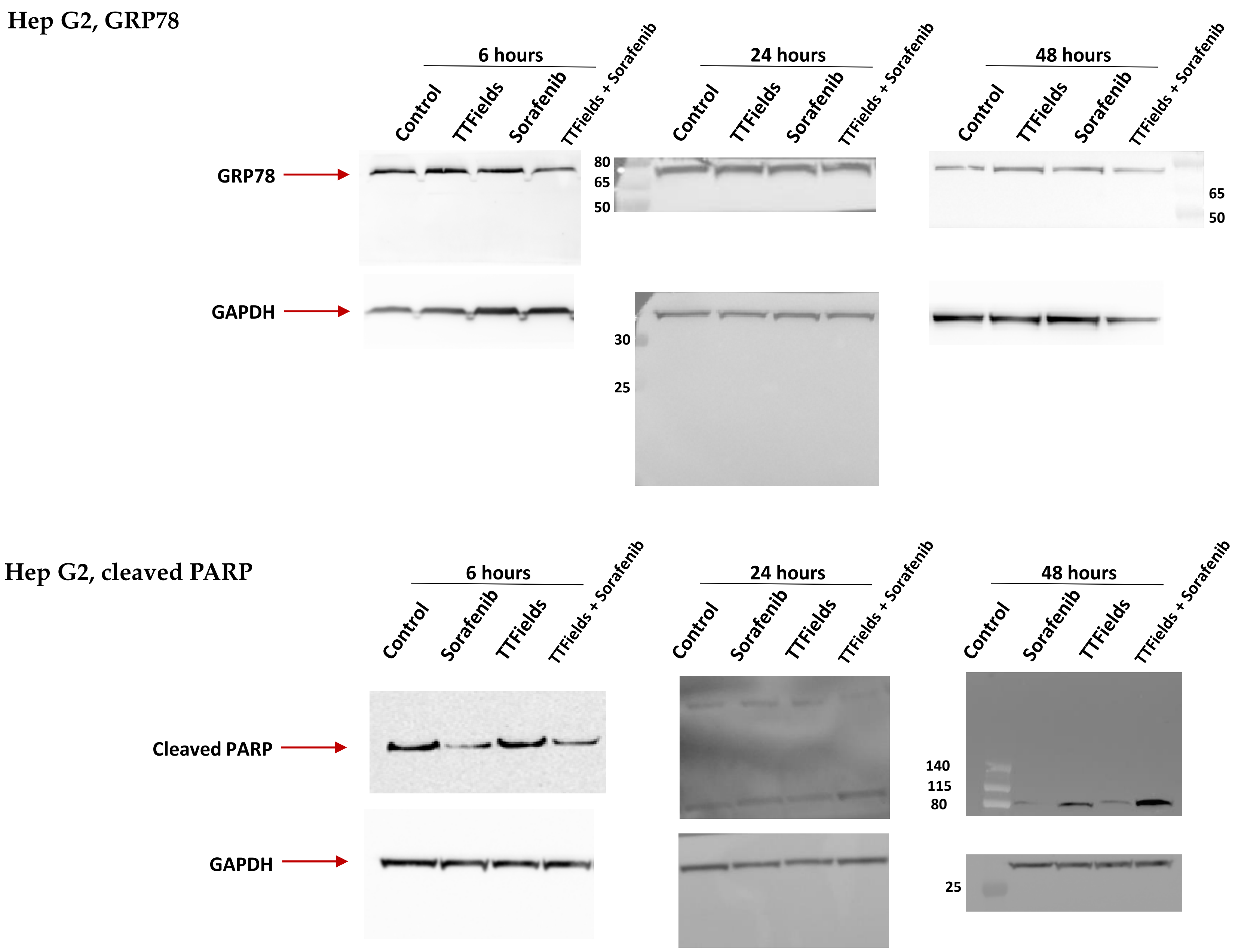
Hep G2, GRP78
6 hours
24 hours
48 hours
Control
TTFields
Sorafenib
TTFields + Sorafenib
GRP78
GAPDH
80
65
50
30
25
65
50
Hep G2, cleaved PARP
6 hours
24 hours
48 hours
Control
Sorafenib
TTFields
TTFields + Sorafenib
Cleaved PARP
GAPDH
140
115
80
25

Figure S1.

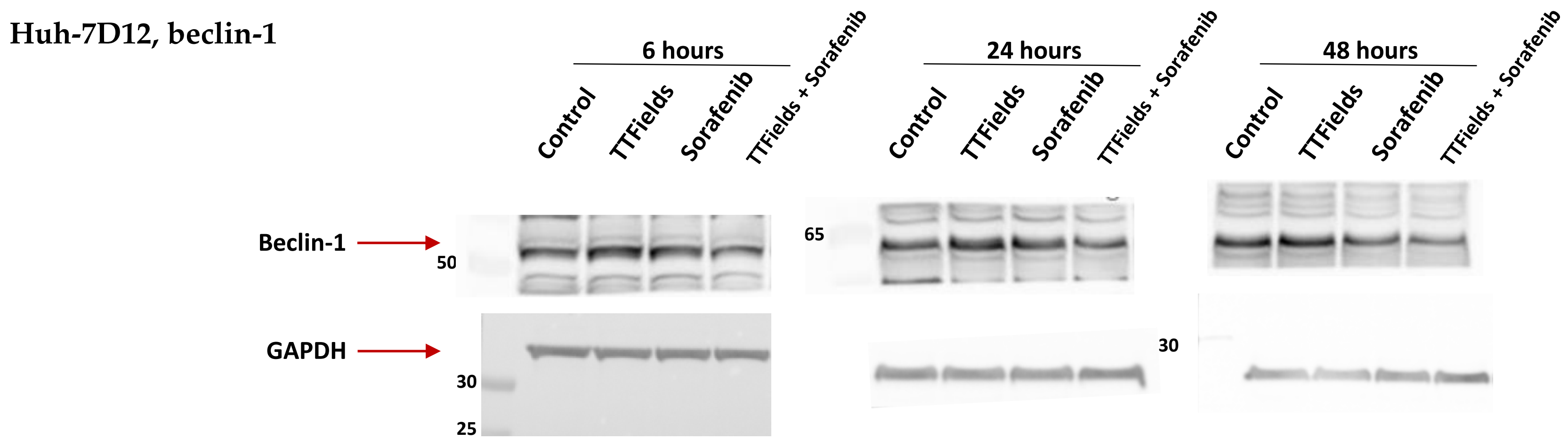
Huh-7D12, beclin-1
6 hours
24 hours
48 hours
Control
TTFields
Sorafenib
TTFields + Sorafenib
Beclin-1
GAPDH
50
65
30
25

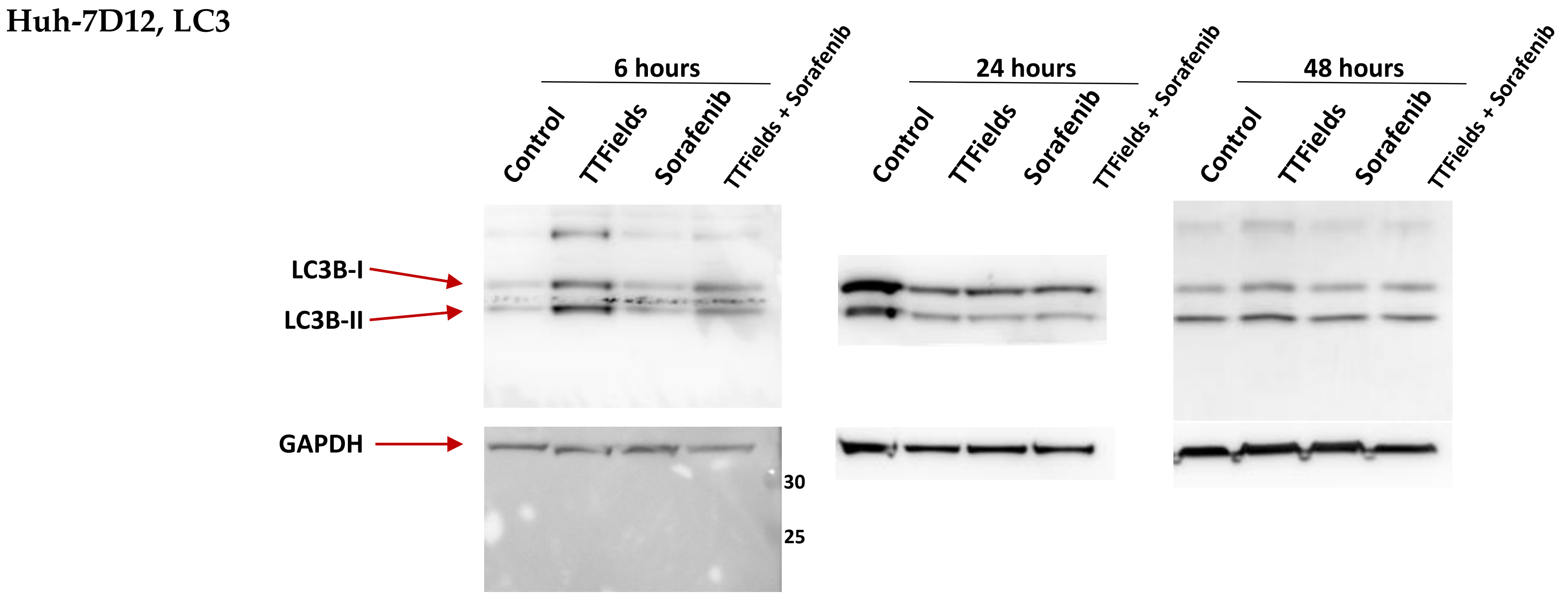
Huh-7D12, LC3
6 hours
24 hours
48 hours
Control
TTFields
Sorafenib
TTFields + Sorafenib
LC3B-I
LC3B-II
GAPDH
30
25

Figure S1.

Huh-7D12, GRP78

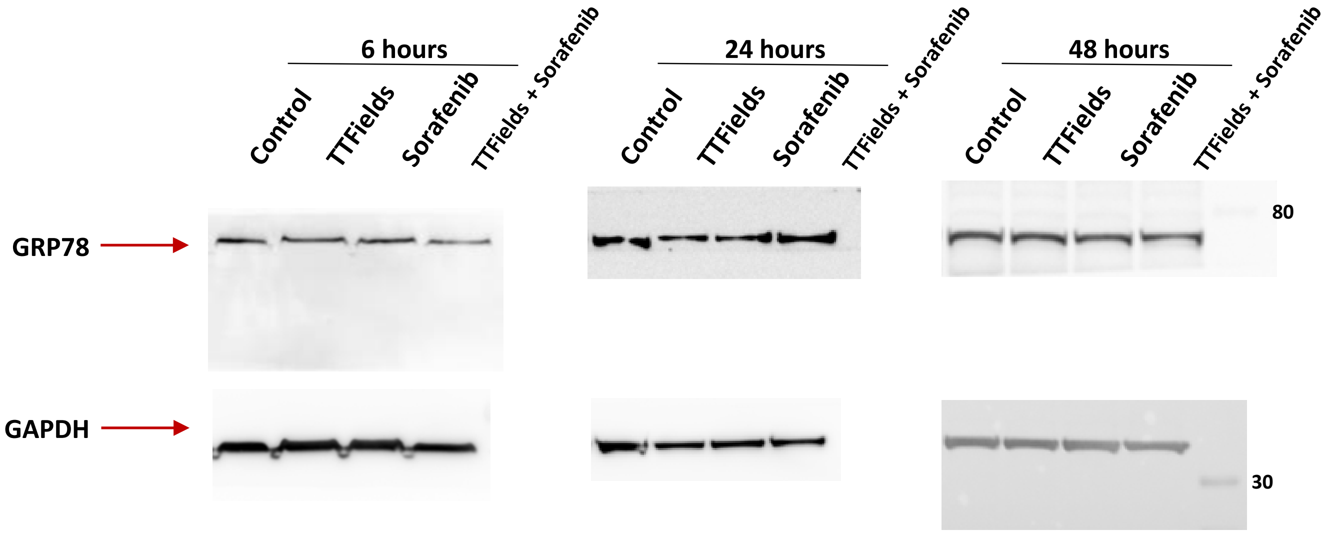

Huh-7D12, cleaved PARP

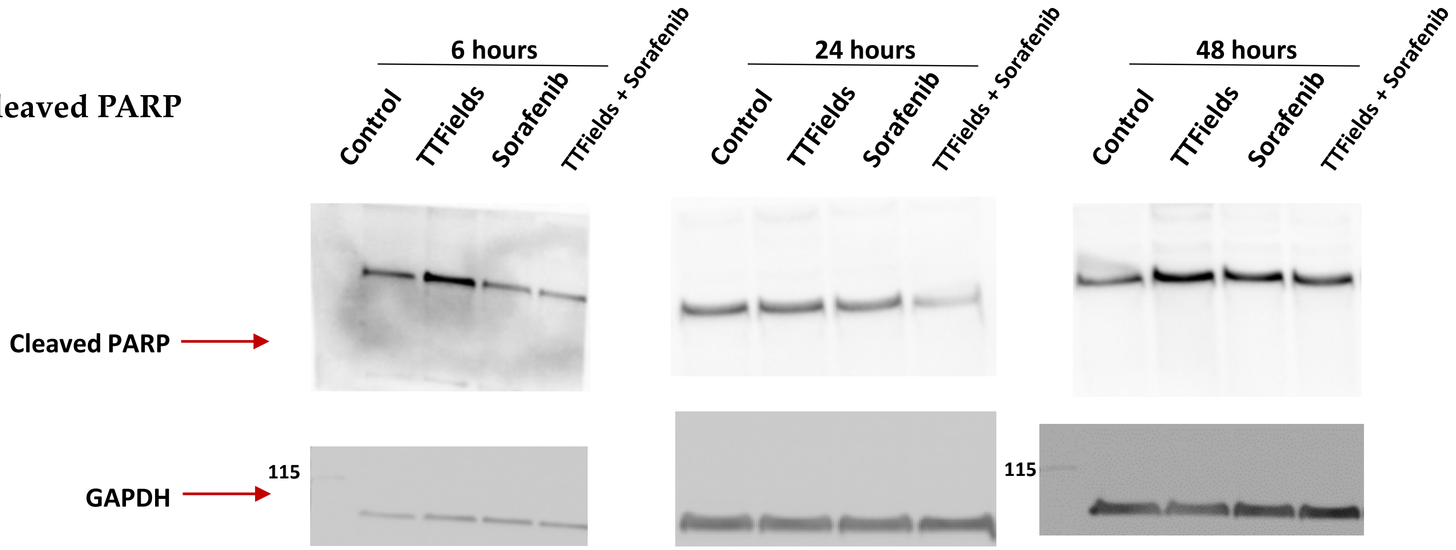

Supplement: Supplementary file 1 [file cancers-14-02959-s001.zip › cancers-1602413-supplementary.pdf]
